# Role of Body Mass Index on Perceived Stress in Medical Students

**DOI:** 10.1192/j.eurpsy.2023.1922

**Published:** 2023-07-19

**Authors:** R. Jbir, R. Masmoudi, M. Abdelkefi, S. Hentati, I. Feki, R. Sellami, J. Masmoudi

**Affiliations:** 1 Psychiatry ‘A’, Hedi Chaker hospital university; 2 Hedi Chaker hospital universiry; 3 Hedi chaker hospital university; 4 Psychiatry ‘A’; 5 psychiatry A, Hedi chaker university hospital, Sfax, Tunisia

## Abstract

**Introduction:**

The study period to become a medical professional is notoriously stressful This label can be attributed to various factors like long tedious training, social expectations, long work hours, high liability, sleep deprivation, and a constant lack of time to relaxin this vulnerable area, physical appearance, either weight loss or obesity, can affect the mental health of these young people.

**Objectives:**

To determine the role of body mass index (BMI) on the levels of perceived stress in medical students.

**Methods:**

Our study was descriptive and analytical cross-sectional, carried out with medical students in the faculty of medicine of sfax (Tunisia) during October 2022.

An anonymous survey was asked to the students.

Data collection was done by a self-administered questionnaire via Google Forms administered in the students’ Facebook groups. The questionnaire was composed of a part for the collection of socio-demographic data and a psychometric scale:

- Cohen’s Perceived Stress Scale (PSS) to determine the level of stress

**Results:**

A total of 95 responses was collected.The average age of our sample was 25.8±3.4 with female predominance (78,9%).

The half of the population (53,7%) were residents in medicine.

The majority of them (88,4%) had an average socio-economic level and singles (83,2%) .

81,1% had a stressor related to studies in 50,5%,to family in 40 % and financial in 9,5%.

Tobacco consumption were reported by 14,7 % .

A psychiatric history was reported by 17.9% of the students, 76.5% of whom are anxiety disorders.

27.4% tried to control their weight. Several methods of weight control were used, the most frequent (65.4%) was diet, none resorted to laxatives and 8.4% consulted a nutritionist.

Almost half of the population (57,9%) slept between 5 and 7 hours.

The average body mass index was 23.64 kg/m2 (SD=3.53).

According to PSS scores, 21.1% of students had severe level of stress, 69.5% had moderate stress level while 9.5% had low level of stress.

Those followed in psychiatry had a higher level of stress (p<10-3), especially those with anxiety disorder (p=0,02).

The students pressed for weight control were more stressed than their peers.

The levels of stress were higher among underweight students (BMI < 18.5) and overweight students (BMI >25) without significantly correlations.

**Conclusions:**

The current study revealed that medical students, especially underweight or overweight students, are more susceptible to develop stress symptoms,that is why psychic support must be available on their university.

**Disclosure of Interest:**

None Declared

